# Applicability of models to estimate traffic noise for urban roads

**DOI:** 10.1186/s40201-015-0240-9

**Published:** 2015-12-02

**Authors:** Ricardo A. Melo, Roberto L. Pimentel, Diego M. Lacerda, Wekisley M. Silva

**Affiliations:** Department of Civil and Environmental Engineering, Federal University of Paraíba, Campus I, Cidade Universitária, 58051-900 João Pessoa, Brazil

**Keywords:** Traffic noise, Models, Urban roads

## Abstract

**Background:**

Traffic noise is a highly relevant environmental impact in cities. Models to estimate traffic noise, in turn, can be useful tools to guide mitigation measures. In this paper, the applicability of models to estimate noise levels produced by a continuous flow of vehicles on urban roads is investigated. The aim is to identify which models are more appropriate to estimate traffic noise in urban areas since several models available were conceived to estimate noise from highway traffic.

**Results:**

First, measurements of traffic noise, vehicle count and speed were carried out in five arterial urban roads of a brazilian city. Together with geometric measurements of width of lanes and distance from noise meter to lanes, these data were input in several models to estimate traffic noise. The predicted noise levels were then compared to the respective measured counterparts for each road investigated. In addition, a chart showing mean differences in noise between estimations and measurements is presented, to evaluate the overall performance of the models. Measured Leq values varied from 69 to 79 dB(A) for traffic flows varying from 1618 to 5220 vehicles/h. Mean noise level differences between estimations and measurements for all urban roads investigated ranged from −3.5 to 5.5 dB(A).

**Conclusions:**

According to the results, deficiencies of some models are discussed while other models are identified as applicable to noise estimations on urban roads in a condition of continuous flow. Key issues to apply such models to urban roads are highlighted.

## Background

Traffic noise has increased and nowadays is a relevant environmental impact in brazilian cities, because the increasing of number of vehicles. The total vehicle fleet increased 118 % from the year 2000 to 2010, whereas motorcycle fleet grew 309 % during the same period [1, 2].

In a review on the subject [3], several aspects of the problem were discussed and it was mentioned that the noise index mostly employed on noise-annoyance relationships is the equivalent sound pressure level *L*_*eq*_.

The A-weighted equivalent sound pressure level (*L*_*eq*_*(A)*) or, alternatively, the percentile levels *L*_*10*_ or *L*_*90*_, are estimated by traffic noise prediction models. The main advantage of models is to estimate noise levels on a much reduced time and cost than by carrying out measurements. Also, it is the sole alternative when planning traffic routes. Due to its peculiar characteristics, noise from traffic in urban roads (city traffic) might potentially differ from that observed in highways.

The aim of this paper is to investigate the adequacy of some models to estimate urban traffic noise, taking as a case study five urban arterial roads of a medium-sized city, in which a continuous traffic flow was observed. The focus is to seek for a combined effect of significant traffic flow and non-congested traffic, so as to represent a critical condition in terms of urban traffic noise. The noise produced in such conditions could be, in principle, estimated by models presented in the literature and based on highway traffic. However, some key issues typical of urban roads such as short distances between road and receiver, traffic composition and speed lower than those observed in highways may interfere. Bearing this in mind, eight traffic noise models were selected so as estimations from these models could be compared to measured values: Golmohammadi et al. [4], Kinsler et al. [5], Birkan et al. [6], CoRTN [7, 8], Tansatcha et al. [9], RLS-90 [10], Calixto et al. [11] and Paz and Zannin [12].

### Features of traffic noise models

The selected traffic noise models are briefly described in this Section; the reader can refer to the respective original works to check the expressions of each model.

#### Golmohammadi et al.

Golmohammadi et al. [4] conceived a model based on parameters including traffic flow and speed, vehicle types and road dimensions, the latter including section length, width and gradient of road, and height of buildings around the road. Measurements were taken at a distance of 3.0 m from the roadside edge, that it is usually compatible with the space available along urban roads. As for the traffic, a condition of free flow in straight road segments of dual carriageways was considered.

#### Kinsler et al.

The model presented by Kinsler et al. [5] considers a straight, two-lane road of infinite length, zero grade and negligible truck traffic, and takes the flow of vehicles and speed as input. The equivalent continuous A-weighted sound pressure level (*L*_*eq*_) in dB(A) is evaluated for a reference distance of 15 m between the evaluation point and centerline of the nearest lane, but it is necessary a correction term for distance greater than 15 m.

Regarding the speed, changes are taken into account, based on a ratio of the input speed and a reference speed of 88 km/h, which is higher than the usual speed on urban roads.

#### Birkan et al.

This model was employed by these authors to estimate traffic noise on urban roads [6]. The sound pressure level in the vicinity of a street segment, *L*_*eq*_, is estimated taking into account the flow of vehicles and speed as input parameters. No explicit reference is made to traffic composition. The noise level is corrected through a term that contains the distance of the sound receptor from road edge plus one third of the width of a lane. A basic correction factor that depends on pavement type is also included.

#### CoRTN

This model is based on an hourly basic noise level *L*_*10*_, in dB(A), for a given hourly traffic flow at a mean speed of 75 Km/h. The *L*_*10*_ is further adjusted to take into account type of vehicles, gradient, road surface and speed.

Regarding the type of surface of the pavement, for impervious bituminous road surfaces, 1 dB(A) should be subtracted from the basic noise level when the traffic speed is below75 Km/h. On the contrary, if traffic speed is equal or greater than 75 Km/h a correction applies, being a function of the texture depth of the road surface [7].

In sequence, further corrections are considered to take into account, as appropriate, the effects of distance from the source line, the nature of the ground surface, and screening from any intervening obstacles.

On sites where the ground surface between the edge of the nearside carriageway and the reception point is totally or partially of an absorbent nature, (e.g. grassland) a correction for ground absorption is obtained from a chart provided [7], which depends on height and distance, and the proportion of absorbing ground.

Recently, updates of the CoRTN model were proposed [8]. Recommendations applicable to this investigation involved adopting the dual source line approach for all dual carriageways, irrespective of the number of lanes per carriageway or the separation of horizontal or vertical alignments. It is worth mentioning that a provision for calculating the noise from separate carriageways was already presented as an option in the CoRTN original model. Also, it was recommended that the heavy vehicle category is redefined as vehicles with unladen weight greater than 3500 kg, instead of the originally adopted unladen weight of 1525 kg.

Finally, since the CoRTN model is based on *L*_*10*_ values, a conversion to *L*_*eq*_ is needed so as to compare these estimates with those from the other models.

#### Tansatcha et al.

Tansatcha et al. [9] considered some vehicle classes: automobile, truck, bus and motorcycle. To start, the noise level for a single vehicle is calculated. Such a noise is considered to be produced in a 10-s time interval. Then, the 1 h equivalent sound pressure level for vehicle class *i* is calculated, as a function of the hourly flow of that vehicle type, and including corrections for ground absorption and distance. Regarding the latter, the reference distance considered is 15 m and the distance of the reception point is taken from the center line of the traffic lane.

By combining the contribution of vehicle classes (herein, automobiles, motorcycles, buses and light trucks were considered), a total hourly equivalent sound level on a single roadway (or lane), namely *L*_*eq*(1h),*j*_ in dB(A), is calculated as the logarithm sum of the contributions of each vehicle type. Finally, the total equivalent sound level in 1 h, *L*_*eq(1h)*_*,* in dBA, is obtained in a similar way by the logarithm sum of the contribution of all lanes.

#### RLS-90

In Germany, a model for road traffic noise estimation is in use, identified as RLS-90 [10], being applicable for a range of traffic flow from 10 to 10,000 veh/h. This model has flexibility in the sense that it considers several correction terms for a wide range of traffic and site conditions. It starts from the A-weighted mean noise level at a distance of 25 m from the centerline of the road and for the following conditions: (*i*) speed of 100 Km/h; (*ii*) non-grooved asphalt road surface; (*iii*) road gradient less than 5 %; and (*iv*) free field propagation. Inputs in this expression are the traffic flow (veh/h) and percentage of trucks with mass above 2800 kg.

In sequence, corrections for speed, road surface type, gradient and absorption characteristics of adjacent building surfaces are added. The correction for speed considers a combination of speeds of cars and trucks. Finally, spreading and air absorption are considered through a correction factor as a function of distance between the receiver and the sound source.

#### Calixto et al.

A model to predict traffic noise from roads inside urban settings was developed by Calixto et al. [11]. The noise emission levels were defined in terms of *L*_*eq*_, *L*_*10*_ and *L*_*90*_ [dB(A)]. As their work also bore in mind to verify the applicability of the RLS-90 model to brazilian road conditions, they adopted the same reference distance of 25 m. They ended up with simple and straightforward expressions to obtain noise emission levels (*L*_*eq*_, *L*_*10*_ and *L*_*90*_), as a function of the traffic flow and percentage of heavy vehicles. It compared well with the evaluation made by the RLS-90 model.

#### Paz and Zannin

Paz and Zannin [12] proposed linear models based on daytime measurements in urban highways. The range of distances from the road in which measurements were taken was from 15 to 25 m, at a height of 1.20 m. Vehicle flow and traffic composition were the model entries. The concept of class intervals was introduced, that is, different expressions were proposed according to the range of values of noise being estimated. Furthermore, the authors presented expressions based on measured and mean values, the latter being based on adjusted data. Herein, the model selected was identified by Paz and Zannin as Model 18 [12], in which L_eq_ values were estimated based on mean values, class intervals and the entries were the light and total vehicle flows.

## Method

### Site selection

The investigated sites are located in João Pessoa, a medium-sized city of about 723 thousands inhabitants in the northeast of Brazil. In João Pessoa, the vehicle fleet increased about twice in the last decade and is predominantly composed by cars and motorcycles; these vehicles are about 97 % of total vehicle fleet.

The urban arterial roads studied were five avenues of high traffic volume and relevance in the city (between parentheses the abbreviation used in the following paragraphs): José Américo de Almeida (JA), Rui Carneiro (RC), Epitácio Pessoa (EP), Hilton Souto Maior (HM), and D. Pedro II (PII).

**Fig. 1:**
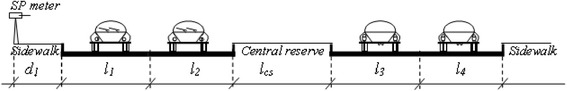
Typical cross section of the sites (not to scale). (Legend: *d*_*1*_: distance between sound pressure level meter (SP meter) and edge of the closest lane; *l*_*i*_: width of *i* ^*th*^ lane (*i*: 1 to 6); *l*_*cs*_: width of central reserve)

All roads had a central reserve and two lanes in each direction, except PII Avenue, with three lanes per direction. The chosen sites to measure noise levels in each avenue had no surrounding buildings or obstacles, being adequate for the application of several noise prediction models. The roads were straight segments, had flat gradients and asphalt mixture surface course. In Fig. 1, a typical cross section of the sites is shown and the relevant dimensions are shown in Table 1. Table 2, in turn, provides information on the ground condition, to apply corrections on calculations, as appropriate. This information was employed to apply corrections due to ground effects for the CoRTN and Tansatcha models.

**Table 1:** Relevant dimensions (in meters) of all sites

Urban road	*l*_*1*_	*l*_*2*_	*l*_*3*_	*l*_*4*_	*l*_*5*_	*l*_*6*_	*l*_*cs*_	*d*_*1*_
JA	3.45	3.45	3.46	3.47	*	*	6.00	1.80
RC	3.50	3.50	3.50	3.50	*	*	1.75	6.90
EP	3.71	3.60	3.60	3.65	*	*	5.45	4.15
HM	3.40	3.50	3.60	3.20	*	*	7.00	9.50
PII	3.50	3.50	3.50	3.50	3.50	3.50	1.00	2.00

Legend- *d*_*1*_ *, l*_*1*_ to *l*_*6*_ and *l*_*cs*_: see legend of Fig. 1

**Table 2:** Features of the ground in all sites

Urban road	*l*_*1*_	*l*_*2*_	*l*_*3*_	*l*_*4*_	*l*_*5*_	*l*_*6*_	*l*_*cs*_	*l*_*s*_
JA	Hard	Hard	Hard	Hard	*	*	Soft	Soft
RC	Hard	Hard	Hard	Hard	*	*	Hard	Hard
EP	Hard	Hard	Hard	Hard	*	*	50 % hard/50 % soft	Hard
HM	Hard	Hard	Hard	Hard	*	*	Soft	Soft
PII	Hard	Hard	Hard	Hard	Hard	Hard	Hard	Hard

Legend- *l*_*1*_ to *l*_*6*_ and *l*_*cs*_: see legend of Fig. 1; *l*_*s*_: width of sidewalk in which the sound level meter was placed

### Measurements of traffic noise

The traffic noise levels were obtained using a class 1 01 dB sound pressure meter model SIP95. Following the procedures recommended in the Brazilian code NBR 10151 [13], the meter was placed 1.2 m above ground, at the sidewalk. The distance from the road varied (see Fig. 1 and Table 1 for the values), due to peculiarities of the sites. The meter was set in fast mode and the microphone was positioned perpendicular to the lane axis. Sites were always in dry condition and absent of other noise sources; temperatures were around 28 °C and the wind was not of attention. The Brazilian code [13] specifies that measurements should be long enough for the noise to be properly characterized, and measurements can be continuous or in intervals.

Measurements in each site were made within a period of 1 h in week days, either during mornings or afternoons, in which there is a combined effect of significant vehicle flow and non-congested traffic. Four records of traffic noise lasting five minutes each was acquired, each representing a quarter of an hour. In order to consider noise fluctuations within the hour, the four acquisitions were separated by intervals of 10 min. As can be seen in Table 3, noise fluctuations within the hour were small and the value of L_eq(1h)_ was obtained by grouping together all measured values.

**Table 3:** Sound pressure levels measured at the sites, dB(A)

Urban road	*L*_*eq, 15 min*_				*L*_*eq(1h)*_	*L*_*10(1h)*_	*L*_*10*_ *-L*_*eq*_
	1st	2nd	3rd	4th			
PII	81.4	77.8	77.0	77.6	78.6	79.1	0.5
JA	72.8	73.2	72.4	73.0	72.9	74.1	1.2
RC	69.9	71.2	70.2	70.0	70.3	72.4	2.1
EP	70.0	73.3	73.0	74.8	73.5	77.6	4.1
HM	69.2	67.8	66.3	71.1	69.0	71.9	2.9

### Traffic count and determination of speed

A handy video camera Sony model DCR-DVD 610 was used to record the flow of vehicles during 1 h in each site. Later on, in the office, vehicle counting was made from video images. Bearing in mind that the videos also displayed the time together with images, they were also employed to obtain the speed of vehicles. So, the speed of selected vehicles was calculated based upon the time interval that a vehicle took to go through a 20-m long segment, identified in the video by two traffic sign cones. This calculation considered that the speed of a given vehicle remained unchanged between the sign cones. An average of the individual speeds of the selected vehicles was carried out for each lane, carriageway, or for the overall traffic, as appropriate.

## Results and discussion

### Sound pressure levels

In Table 3, a summary of measured equivalent continuous A-weighted sound pressure levels (*L*_*eq(1h)*_) and *L*_*10*_ are presented. The difference between L_10_ and L_eq_ for each site was specifically employed for the conversion of estimations using the L_10_-based CoRTN model to L_eq_, so as to compare with other models.

### Speed, vehicle type and flow

These data are shown in Table 4, in terms of flow of vehicles (per lane, per vehicle type and total), and mean speed per vehicle type.

**Table 4:** Summary of traffic data for all sites

Urban road	JA		RC		EP		HM		PII	
Lane	*Q*	*v*	*Q*	*v*	*Q*	*v*	*Q*	*v*	*Q*	*v*
1	537	61.4	912	45.7	312	61.0	611	70.2	1043	50.5
2	459	74.4	524	61.4	543	63.3	220	82.8	1255	60.4
3	304	65.1	759	64.3	500	59.8	399	76.1	875	68.0
4	318	64.7	836	61.3	406	59.8	427	76.1	602	67.9
5	*	*	*	*	*	*	*	*	891	68.1
6	*	*	*	*	*	*	*	*	554	60.3
Total	1618	*	3031	*	1761	*	1657	*	5220	*
	*q*	%	*q*	%	*q*	%	*q*	%	*q*	%
*q*_*aut*_	1146	70.8	2258	74.5	1250	71.0	906	54.7	3439	65.9
*q*_*uti*_	229	14.2	343	11.3	230	13.1	244	14.7	605	11.6
*q*_*mot*_	205	12.7	345	11.4	189	10.7	399	24.1	987	18.9
*q*_*bus*_	16	1.0	43	1.4	60	3.4	27	1.6	73	1.4
*q*_*tru*_	22	1.4	42	1.4	32	1.8	81	4.9	116	2.2

*Notes*: 1) Flow of vehicles (*Q*, veh/h); mean speed (*v*, Km/h); 2) Flow of vehicle type *i*, *q* (*aut* = automobiles; *uti* = utility vehicles; *mot* = motorcycles; *bus* = buses; *tru* = trucks)

The results presented in Table 4 show that the highest flow of vehicles was on PII Avenue, with 5220 veh/h, followed by RC Avenue, with 3031 veh/h. These avenues link populated neighborhoods to downtown, in which commercial centers, public administration and public services are present. In the other three avenues, a flow of vehicles about 1700 veh/h can be observed.

Furthermore, it can also be seen that automobile, motorcycle and utility vehicles were predominant, corresponding to about 96 % of the traffic volume. Also, it may be observed that motorcycles are a non-neglibible part of the total flow, reaching 24 % of traffic volume in one of the roads. Presence of motorcycles is a trend that is expected to increase since sales of motorcycles increased about 300 % in the last 10 years.

For speed, the values were in the broad range from 46 to 83 km/h, considering the traffic flow per lane. However, most values were between 60 and 70 km/h.

### Determination of noise level by the models

Estimations considered the flow of vehicles per lane, per direction and total, as applicable to each model.

It was considered that a tolerable difference between estimated and measured values can be up to 1.5 dB. This is equivalent to a difference of 1 dB to take into account the accuracy of the sound pressure meter, plus an estimating error of ± 0.5 dB, which is a negligible change of sound pressure level perceptible by a human being.

**Fig. 2:**
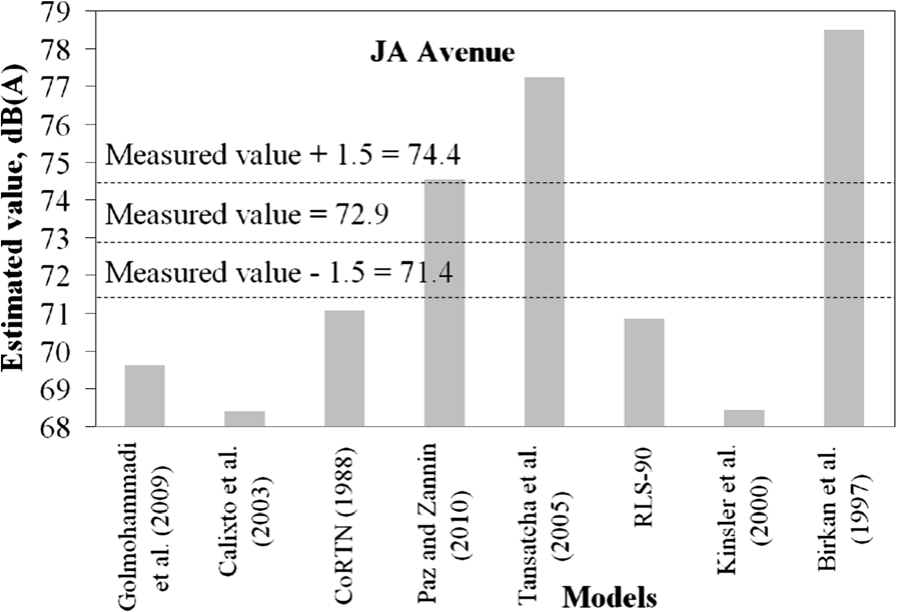
Measured *L*_*eq*_ interval and estimated *L*_*eq*_ by models for JA avenue

**Fig. 3:**
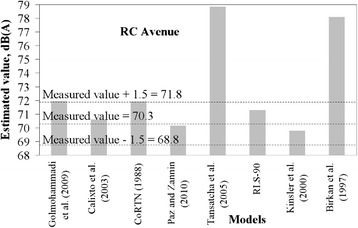
Measured *L*_*eq*_ interval and estimated *L*_*eq*_ by models for RC avenue

**Fig. 4:**
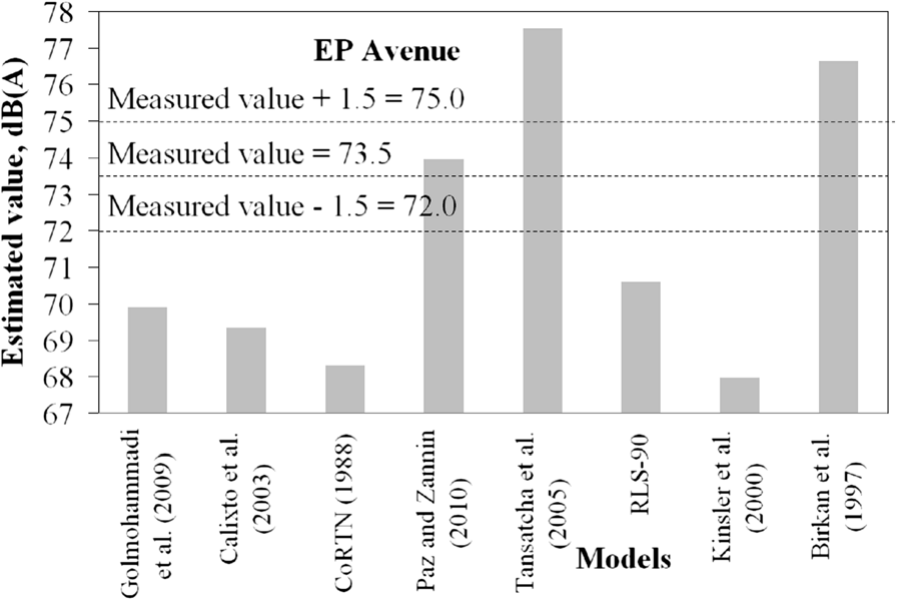
Measured *L*_*eq*_ interval and estimated *L*_*eq*_ by models for EP avenue

**Fig. 5:**
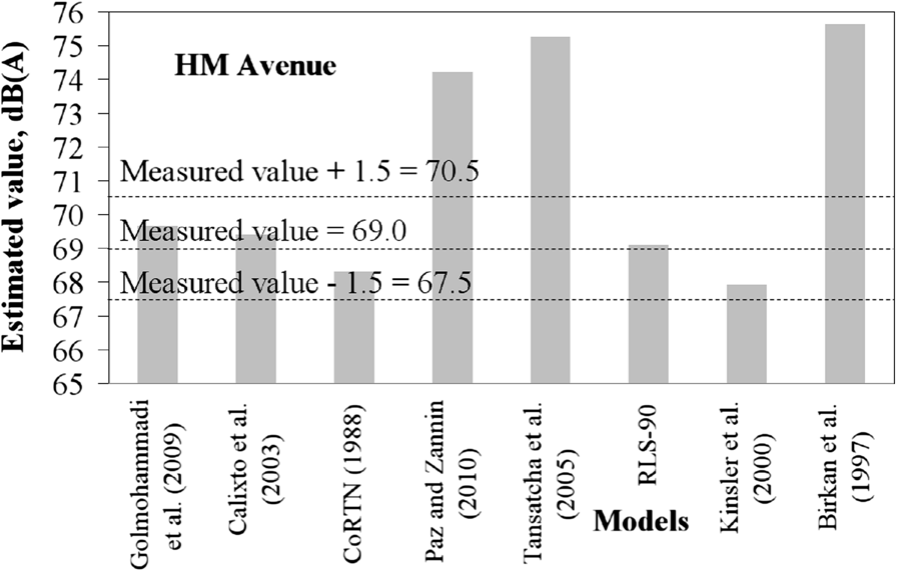
Measured *L*_*eq*_ interval and estimated *L*_*eq*_ by models for HM avenue

**Fig. 6:**
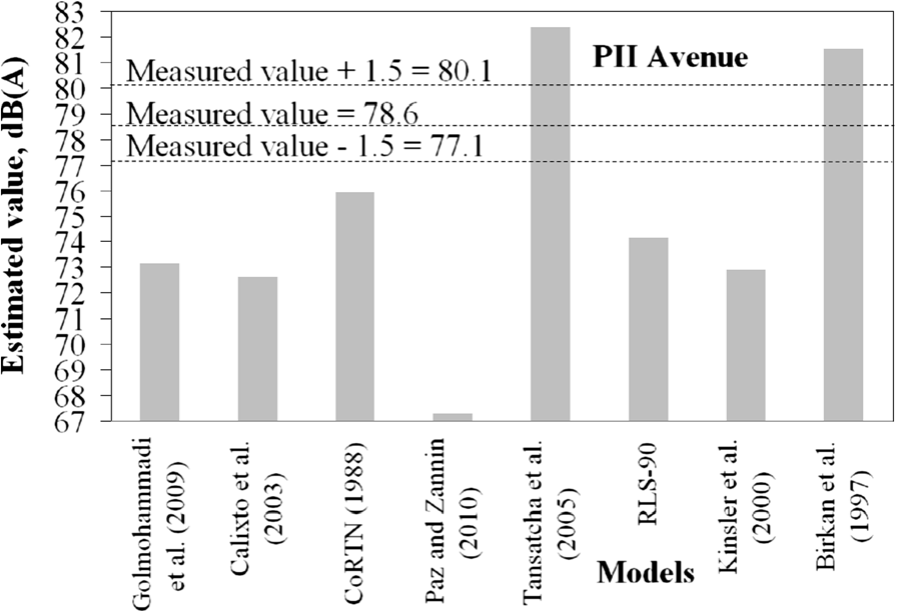
Measured *L*_*eq*_ interval and estimated *L*_*eq*_ by models for PII avenue

Figures 2, 3, 4, 5 and 6 present estimated values of *L*_*eq*_*(A)* by the aforementioned models, together with the measured values.

It is not expected that a given model always provides estimation within the predefined margin of tolerance from the measured value. A significant number of variables and specific road conditions of a location may impair estimations. However, general trends can be observed, and from these some findings are highlighted:

- There is a tendency presented by the Kinsler et al. [5] and Calixto et al. [11] models in underestimating noise levels. It should be noted that in Kinsler et al. [12], no correction of noise levels for distances less than 15 m is adopted whereas in Calixto et al. [11] model, the reference distance is 25 m and no correction is considered for distance. As for the latter, the estimations agreed well with those from the RLS-90 model for a distance of 25 m [11]. It is, thus, worth mentioning that models to estimate noise in urban areas require correction terms for shorter distances than those adopted by such models, as observed in the sites in which measurements were taken. Therefore, this may be appointed as a reason why these two models underestimated the noise levels;
- Sound pressure levels estimated by Birkan et al. [6] and Tansatcha et al. [9] models were higher than others on all sites. With regard to Tansatcha et al. [9] model, a time interval of 10 s was defined to evaluate the noise level for each vehicle, and this limits the flow to 360 veh/h. This implies that this model would overestimate noise levels for vehicle flows per lane above such values, as it occurred in several lanes of the investigated sites. With regard to the model by Birkan et al. [6], the reason why it overestimated the noise is not clear since the information describing the model is limited. One point to call attention is that the traffic composition (that is, the percentage of heavy vehicles) is not taken into account explicitly in the expression of that model. Therefore, the model was possibly adjusted to a specific traffic composition that may differ from those observed in the measurement sites.
- Results for the PII avenue (Fig. 6) shows that a combination of very high traffic volume and a very short distance from the sound level meter to the road edge (due to restrictions on site) led to an underestimation of the traffic noise by the majority of the models that presented good estimates for the other sites.

**Fig. 7:**
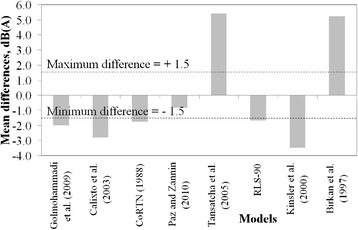
Summary of mean noise level differences between estimations and measurements for all urban roads

Figure 7 provides a synthesis of the results for the five avenues, in terms of the mean difference between each estimation and respective measured value. The models of Golmohammadi et al. [4], CoRTN [7], RLS-90 [10] and Paz and Zannin [12] presented a good performance, the latter being within the specified range of the experimental values.

## Conclusions

The aim of this paper was to investigate the adequacy of some noise level models in estimating urban traffic noise in roads of continuous traffic flow. The following models were chosen: Golmohammadi et al. [4], Kinsler et al. [5], Birkan et al. [6], CoRTN [7], Tansatcha et al. [9], RLS-90 [10], Calixto et al. [11] and Paz and Zannin [12]. Measurements of in situ noise levels, flow of vehicles, speed, and road layout were carried out for five urban arterial roads. In sequence, this database was used as input of the models. Then, measured and estimated noise levels were compared to analyze the applicability of the models in estimating the urban traffic noise.

The CoRTN [7], Golmohammadi et al. [4], RLS-90 [10] and Paz and Zannin [12] models showed the best performance, having the lowest mean difference to the measured noise levels. These best performances can be related to some features of urban roads covered by these models, such as flexibility in defining traffic composition, corrections of spreading or else reference distances compatible to urban settings and compatible ranges of speed and flow. Such models resulted potentially suitable to estimate noise from continuous traffic flow in urban arterial roads with similar traffic features and road layout of the roads investigated here.

Although Calixto et al. [11] model was also developed from measurements in urban roads, the predicted noise levels were not so good. This may be attributed to the absence of corrections for distance, associated to a reference distance of 25 m adopted by this model (to make it intentionally compatible with the RLS-90 model), leading to underestimations when applying this model to the short distances observed at the sites. Kinsler et al. [5] presented the same problem, in the sense that it does not support corrections for distances below 15 m. Finally, for the model from Tansatcha et al. [9], it is apparent that the overestimation of the estimations was related to an upper limit on the flow of vehicles, this being inherently associated to the way the model was conceived. The flows on the investigated sites were much higher than such a limit; this way, when a high flow is input into the model, it results in overestimation.
